# Metagenome Sequencing of the Greater Kudu (*Tragelaphus strepsiceros*) Rumen Microbiome

**DOI:** 10.1128/genomeA.00897-15

**Published:** 2015-08-13

**Authors:** Anita N. Dube, Freeman Moyo, Zephaniah Dhlamini

**Affiliations:** Department of Applied Biology and Biochemistry, National University of Science and Technology, Bulawayo, Zimbabwe

## Abstract

Ruminant herbivores utilize a symbiotic relationship with microorganisms in their rumen to exploit fibrous foods for nutrition. We report the metagenome sequences of the greater kudu (*Tragelaphus strepsiceros*) rumen digesta, revealing a diverse community of microbes and some novel hydrolytic enzymes.

## GENOME ANNOUNCEMENT

Metagenomics enables the direct genetic analysis of genomes contained within an environmental sample as a unit without cultivating individual members (1, 2). Ruminants are ungulate mammals with a multichambered stomach. Their first stomach, the rumen, contains a complex array of microbes that assist these animals to derive nutrients and energy from relatively poor vegetation. Rumen microorganisms, especially from wild animals, are an important source of novel industrially important enzymes. Wild animals of the African savanna, like the greater kudu (*Tragelaphus strepsiceros*), utilize dry plant material, which is high in cellulose content (3), better than domesticated animals, and hence it is assumed that wild ruminants may have greater diversity of novel microbes than their domesticated counterparts. Identification and molecular characterization of these microbes is of paramount importance for bioprospecting endeavors (4). Here, we present the whole-genome shotgun (WGS) metagenome sequences of the greater kudu rumen contents.

Whole rumen digesta was collected from an adult male kudu slaughtered on a game farm situated in the Bubi District near Bulawayo, Zimbabwe. The digesta was taken to the laboratory, where it was portioned into the liquid, fiber-adherent, and fiber-associated microbe fractions using standard methods (5, 6). These microbe fractions were pooled and used to extract metagenomic DNA using the Epicenter Meta-G-Nome DNA isolation kit (Madison, WI, USA).The metagenomic DNA was subjected to WGS at Inqaba Biotech Laboratories in Pretoria, South Africa, on an Illumina MiSeq sequencing platform by generating paired-end reads from libraries with 250-bp inserts. The sequences were analyzed at the European Bioinformatics Institute (EBI) of the European Molecular Biology Laboratory (EMBL) using the EBI Metagenomics Analysis Pipeline version 1 (7).

The sequencing procedure produced 1,057,478 reads, of which 944,362 passed the quality control parameters of the analysis pipeline. Taxonomic analysis on 1,275 identified operational taxonomic units (OTUs) showed that 95.8% of the sequences were of bacterial origin, 3.8% were unassigned, and 0.3% were archaea. At the phylum level, 39.2% of the reads were assigned to *Firmicutes*, 21.6% to unassigned bacteria, 18.4% to *Bacteroidetes*, 4% to *Verricomicrobia*, and 3.8% to *Proteobacteria*. Among the *Firmicutes*, 90% were related to the genera within the class *Clostridia*, of which the predominant families were *Lachnospiraceae* (28%), *Veillonellaceae*, (14%), and *Ruminococcaceae* (10%). The observed taxonomic distribution is consistent with the current understanding of rumen microbiota composition (8, 9).

Functional analysis was done on 930,067 reads with predicted coding sequences. Of these only 396,910 had InterPro matches. Of interest were notable carbohydrate metabolic processes and general hydrolase activities, which were found to be 6.2% of biological processes and 13.1% molecular functions, respectively. The data generated from this kudu rumen metagenome add valuable information for future studies, particularly to those relating to isolation of novel enzymes such as cellulases, which can be used in the production of biofuels.

### Nucleotide sequence accession number.

This whole-genome shotgun metagenomic project has been deposited in the European Nucleotide Archive at EMBL-EBI under the accession number ERR688806.

## References

[1] Thomas T, Gilbert J, Meyer F 2012 Metagenomics—a guide from sampling to data analysis. Microb Inform Exp 2:3. doi:10.1186/2042-5783-2-3. 22587947 PMC3351745

[2] Sabree ZL, Rondon MR, Handelsman J 2009 Metagenomics, p 622–633. *In* Schaechter M (ed), Encyclopedia of microbiology, 3rd ed. Amsterdam, the Netherlands, Elsevier Academic Press.

[3] Boomker EA 1984 Seasonal variation of *in vitro* digestibility in the kudu, *Tragelaphus strepsiceros*. Can J Anim Sci 64:208–209. doi:10.4141/cjas84-227.

[4] Das KC, Qin W 2012 Isolation and characterization of superior rumen bacteria of cattle (*Bos taurus*) and potential application in animal feedstuff. Open J Anim Sci 2:224–228. doi:10.4236/ojas.2012.24031.

[5] Sebastian R, Kim J, Kim T, Lee K 2013 Metagenomics: a promising approach to assess enzyme biocatalysts for biofuel production. Asian J Biotechnol 5:33–50. doi:10.3923/ajbkr.2013.33.50.

[6] Whitehouse NL, Olson VM, Schwab CG, Chesbro WR, Cunningham KD, Lykos T 1994 Improved techniques for dissociating particle-associated mixed ruminal microorganisms from ruminal digesta solids. J Anim Sci 72:1335–1343. 8056682 10.2527/1994.7251335x

[7] Hunter S, Corbett M, Denise H, Fraser M, Gonzalez-Beltran A, Hunter C, Jones P, Leinonen R, McAnulla C, Maguire E, Maslen J, Mitchell A, Nuka G, Oisel A, Pesseat S, Radhakrishnan R, Rocca-Serra P, Scheremetjew M, Sterk P, Vaughan D, Cochrane G, Field D, Susanna-Assunta SSA 2014 EBI metagenomics—a new resource for the analysis and archiving of metagenomic data. Nucleic Acids Res doi:10.1093/nar/gkt961. PMC3965009 24165880

[8] Chaucheyra-Durand F, Ossa F 2014 Review: the rumen microbiome composition, abundance, diversity, and new investigative tools. Prof Animal Sci 30:1–12.

[9] Kim M, Morrison M, Yu Z 2011 Status of the phylogenetic diversity census of ruminal microbiomes. FEMS Microb Ecol 76:49–63. doi:10.1111/j.1574-6941.2010.01029.x. 21223325

